# Primary malignant non-Hodgkin’s lymphoma of the breast: a study of seven cases and literature review

**DOI:** 10.1186/1477-7819-10-151

**Published:** 2012-07-16

**Authors:** Bourhafour Mouna, Boutayeb Saber, El Harroudi Tijani, M’rabti Hind, Taleb Amina, Errihani Hassan

**Affiliations:** 1 Department of Medical Oncology, National Institute of Oncology, Rabat, 10000, Morocco; 2 Faculty of Medicine, Oujda, Morocco, Department of Surgery, National Institute of Oncology, Rabat, 10000, Morocco

## Abstract

**Introduction:**

Primary breast lymphoma is an uncommon disease with poor clinical outcome. Breast lymphomas present less than 0.5% of malignant breast neoplasms and 2.2% of extranodal lymphomas. This study investigated the clinicopathological features and optimal treatment of PBL.

**Case presentations:**

Clinical records of seven Moroccan PBL patients, treated at the National Institute of Oncology, Rabat, Morocco, from 2002 to 2010, were reviewed. Six of the patients were women and one a man, with ages ranging from 32 to 76. Five patients had stage IE and two stage IIE. All of the patients were classified with DLBCL. Of seven patients, one received a mastectomy and three excision of the breast lesion. Axillary dissection was performed in three patients. Two patients received chemotherapy followed by radiotherapy, while four received chemotherapy alone. Complete remission (CR) following primary treatment for all patients with PBL except in two cases was obtained. In one patient, recurrence occurred.

**Conclusions:**

There is no consensus on the question of how to best treat PBL: Mastectomy offers no benefit in the treatment of PBL. The combined therapy approach, with chemotherapy and radiotherapy, is the most successful treatment. PBL is poorly represented in rituximab-containing trials in DLBCL patients; there is not much experience with this agent in breast DLBCL. Because of the high incidence of central nervous system (CNS) involvement in PBL patients, many authors strongly believe that patients with aggressive forms of PBL should receive CNS infiltration prophylaxis.

## Background

Primary malignant lymphoma of the breast (PLB) appears to be a rare disease, and few clinicopathological features of the disease have been discussed in prior studies. It accounts for 2.2% of extranodal lymphomas and constitutes 0.04% to 0.5% of malignant breast neoplasms [1]. The frequency with which the various histopathological types occur is difficult to determine, because terminology varies among the relatively small cases series that have been reported. Despite the clinical and radiographic similarities between breast lymphoma and carcinoma, the prognosis, as reported in the literature, varies, as do the applied treatment modalities, which include surgery, radiotherapy and chemotherapy used alone or in combination.

We retrospectively studied seven cases of PLB of the breast seen in patients attending the National Institute of Oncology in Rabat, Morocco, between 2002 and 2010, in an attempt to determine the common clinical features, therapy and prognosis of primary breast lymphoma.

## Patients and methods

### Clinical data

We report a retrospective study in which the database of the Department of Medical Oncology at the National Institute of Oncology in Rabat was searched for cases of breast lymphoma diagnosed over an 8-year period (2002–2010). A total of seven cases with a diagnosis of NHL of the breast were enrolled according to the criteria established by Wiseman and Liao: (1) a pathological specimen with a close association between the lymphomatous infiltrate and the breast tissue; (2) no evidence of widespread disease or prior extramammary lymphoma; (3) the breast is the principal site of involvement, but ipsilateral axillary lymph node involvement is acceptable if both lesions develop simultaneously [2]. Diagnosis was made with a core needle or an excisional biopsy. Histopathological diagnosis was based on the WHO nomenclature [3]. All patients were staged by computed tomography scans of the chest, abdomen and pelvis, and bone marrow biopsy. The staging was based on the Ann Arbor staging system [4].

### Consent and statement of ethical approval

The treatment of each patient was decided by the medical staff of the center, and oral consent was obtained from the subjects. The study was approved by the institutional review boards of the National Institute of Oncology, Cancer Centre in Rabat. This study was approved by the institutional review boards of the National Institute of Oncology in Rabat.

## Results

### Clinical features

The patients’ characteristics are detailed in Table 1. Of the seven patients, six were women and one was a man. The median follow-up time was 36 months (range 12–60). The median age at presentation was 50 years (range 32–76 years). None of the patients had a previous history of benign or malignant breast disease. Four patients had left-sided and two had right-sided disease; one patient presented bilateral involvement of the breast. The initial chief complaint in each case was a sign of a tumor mass, a mass with local inflammation and palpable lymph nodes. “B” symptoms (10% weight loss, night sweats, fever) were reported in five patients. The stage was IE in five patients and IIE in two patients.

**Table 1:** Clinical characteristics of patients with primary non-Hodgkin’s lymphoma of the breast

**Case no.**	**Age/sex**	**Size of tumor (cm)**	**Site of tumor**	**Clinical information**	**Ipsilateral lymph nodes**	**Histology**	**Ann/Arbor**	**IPI**
1	32/F	3	Left lower outer quadrant	Palpable mass	Present	DLBCL	IIBE	1
2	38/F	7	Left	Palpable mass	Absent	DLBCL	IBE	1
3	47/F	4	Right upper outer quadrant	Palpable mass	Present	DLBCL	IIAE	2
4	60/F	4	Right upper outer quadrant	Palpable mass	Absent	DLBCL	IBE	2
5	55/F	-	Left	Palpable mass	--	DLBCL	IBE	2
6	45/F	5 right/3 left	Bilateral	Palpable masses in both breasts	Present (bilateral)	DLBCL	IIAE	4
7	76/M	-	Left	Palpable lumps	Absent	DLBCL	IBE	1

*F* female, *M* male, *L* left, *R* right, *DLBCL* diffuse large B cell lymphoma, *IPI* International Prognostic Index.

### Histological classification

**Figure 1:**
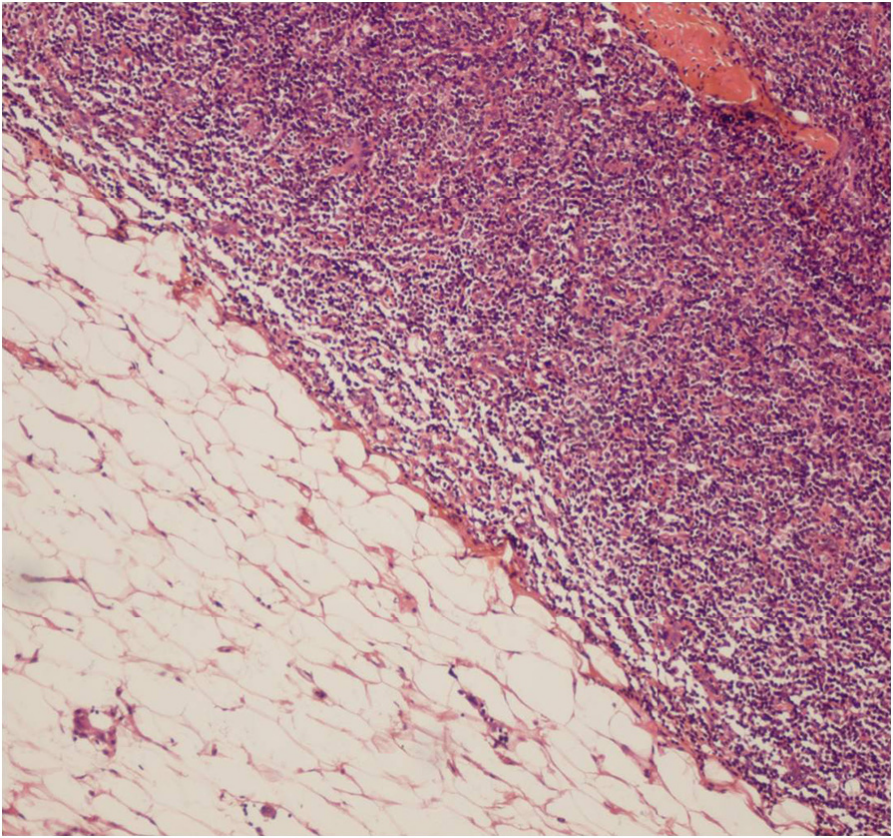
**Diffuse large B-cell lymphoma.** Histologically, the tumor is composed of lymphoma cells (hematoxylin– eosin stain).

**Figure 2:**
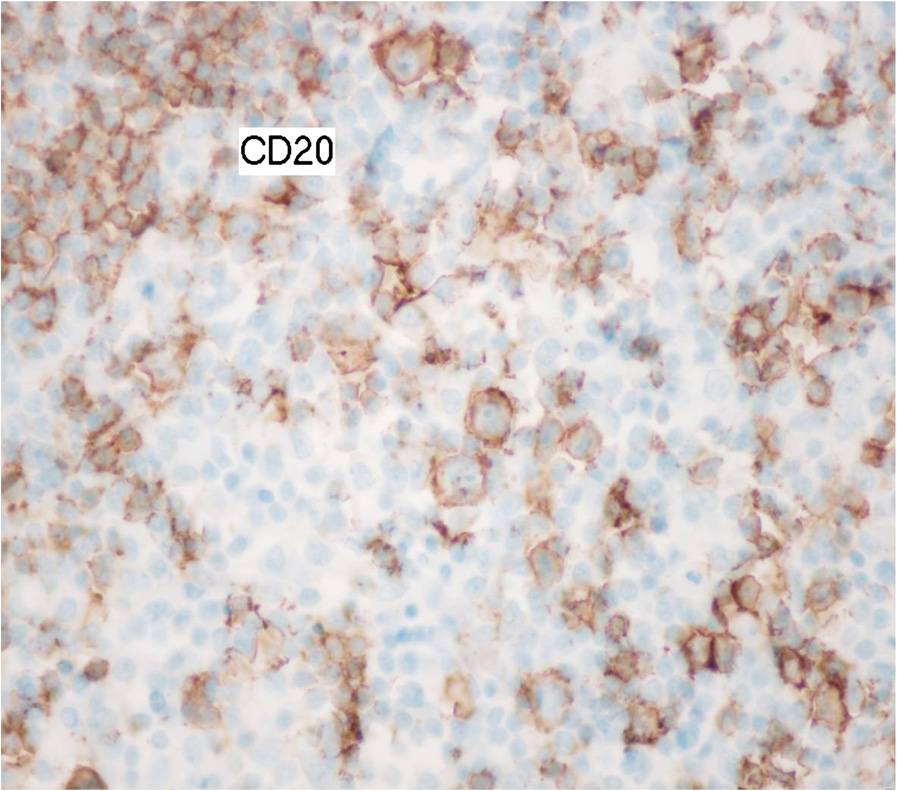
Tumor cells indicate a positive immunohistochemical reaction with CD20.

Histology revealed diffuse large B-cell lymphoma in all cases (Figures 1 and 2).

### Treatment

Three patients had a wide excision of the breast lesion (one quadrantectomy and two tumorectomies); one patient had a mastectomy; the other patients underwent diagnostic biopsies. Axillary dissection was performed in three patients.

Two patients (case 1, 4) with DLBCL received chemotherapy followed by radiotherapy, while four received chemotherapy alone (cases 2, 3, 5 and 6). Anthracycline-containing regimens were administered as the initial systemic chemotherapy for six patients with PBL. The chemotherapy regimen usually included cyclophosphamide, doxorubicin, vincristine and prednisone (CHOP). In two cases, CHOP chemotherapy was combined with rituximab (CHOP-R). The total radiation dose was usually 40 Gy (range, 38 to 45 Gy) to the breast alone. The radiation dose delivered ranged from 36 Gy to 50 Gy directed to the breast and axilla alone.

Case 3 was interesting. The patient was first diagnosed with PNET. Thereafter, she was treated with surgery followed by a regimen of 5 FU and adriamycin. Treatment was completed in eight cycles, and CR was obtained. The patient then presented with a mass in the upper outer quadrants of the right breast 2 years after the diagnosis. Histopathological examination of the biopsy specimen revealed DLBCL of the breast. The patient received combination therapy of adriamycin, cyclophosphamide and vincristine (CHOP) every 3 weeks followed by ICE (ifosfamide, carboplatin and etoposide). CR was achieved after the completion of six cycles of chemotherapy. The patient had no specific symptoms and remained in remission during a follow-up of 24 months.

### Survival

Complete remission (CR) following primary treatment for all patients with PBL except for cases 6 and 7 was obtained. Recurrence occurred in one patient.

Case 6 died during the second cycle of R-CHOP chemotherapy because of febrile neutropenia.

Case 7 received no further treatment because of age and co-morbid conditions. He died of progressive disease 3 months after surgery.

With a follow-up ranging from 12 to 60 months (median; 36 months), four out of seven of our patients are alive and free of disease.

Treatment and follow-up of patients with primary non-Hodgkin’s lymphoma of the breast are described in Table 2.

**Table 2:** Treatment and follow-up of patients with primary non-Hodgkin’s lymphoma of the breast

**Case no.**	**Age/sex**	**Size of tumor (cm)**	**Site of tumor**	**Clinical information**	**Ipsilateral lymph nodes**	**Histology**	**Ann/Arbor**	**IPI**
1	32/F	3	Left lower outer quadrant	Palpable mass	Present	DLBCL	IIBE	1
2	38/F	7	Left	Palpable mass	Absent	DLBCL	IBE	1
3	47/F	4	Right upper outer quadrant	Palpable mass	Present	DLBCL	IIAE	2
4	60/F	4	Right upper outer quadrant	Palpable mass	Absent	DLBCL	IBE	2
5	55/F	-	Left	Palpable mass	--	DLBCL	IBE	2
6	45/F	5 right/3 left	Bilateral	Palpable masses in both breasts	Present (bilateral)	DLBCL	IIAE	4
7	76/M	-	Left	Palpable lumps	Absent	DLBCL	IBE	1

*CHOP* cyclophosphamide, doxorubicin, vincristine, prednisone; *R* rituximab; *ICE* ifosfamide, carboplatin and etoposide; *CR* complete remission; *AW* alive and well.

## Discussion

PBL is a rare, potentially curable, disease and has been considered a distinct clinicopathological entity. PBLs have a reported incidence of 0.04–0.5% of all breast malignancies [5]. PBLs account for less than 1% of all patients with NHLs and approximately 1.7% of all extranodal NHLs [1,5]. At our institution, for the past 8 years, we have treated seven patients for breast involving lymphoma. The term “primary breast lymphoma” (PBL) is used to define malignant lymphomas primarily occurring in the breast in the absence of previously detected lymphoma localizations. Wiseman and Liao [2] are credited with first defining the clinical criteria for the classification of PBL.

With the exception of the recently published prospective Mexican trial (Aviles) and the large study of the International Extranodal Lymphoma Study Group (IELSG) with genuine PBLs and sufficient follow-up, which identified 204 patients [6], in the literature there were only retrospective studies with a relatively limited number of patients that have provided some interesting information [7-11].

The usual clinical feature of a breast lymphoma is a rapidly expanding, painless mass [11,12]. There is a slight predilection for the right breast, but the explanation for this remains unclear [12]. The clinical presentation of our patients with breast lymphoma is similar to that reported by others. Although one of our patients developed bilateral lymphomas of the breast, an incidence of 6% to 13% has been reported [13].

In our study, the median age was 50 years, which is comparable to the 5th decade of life published by other authors [7-11,14,15].

PBL is extremely rare in males, occurring in 1 of our 7 patients, 1 of the 23 patients from the MD Anderson Cancer Center, 1 of the 25 patients from the Mayo Clinic [12,16] and none in the other series [9,17].

All histological types of lymphoma have been described. Primary breast lymphomas are most commonly B-cell lymphomas; approximately one-half are diffuse large B-cell lymphoma. Indolent histologies, follicular non-Hodgkin’s lymphoma or extranodal marginal zone (MALT) lymphoma occur less commonly [7-11,14-17]. All of our cases also had DLBCL. Disagreement regarding the treatment of such disease stems from its rareness, with small case reports; consequently, randomized controlled trials or clinically controlled trials cannot be carried out. The prognosis varies, as do the applied treatment modalities, which include surgery, radiotherapy and chemotherapy used alone or in combination.

Mastectomy has been a common component of PBL therapy for decades and remains a frequent treatment choice in some reports. Several studies found that mastectomy offered no benefit in the treatment of primary breast lymphoma [14,18]. Ideally, surgery should be limited to a biopsy to establish the correct histological diagnosis, leaving the treatment with curative intent to radiotherapy and chemotherapy. In our series, mastectomy was performed in only one patient. Three patients had a wide excision of the breast lesion (one quadrantectomy and two tumorectomies).

Jennings et al. [19] reviewed all published PBL reports from 3 decades (1972–2005). Patient demographics, such as survival, recurrence and time to follow-up, were recorded, in addition to surgical, radiation and/or chemotherapeutic treatment(s). A total of 465 patients were found. The age range was 17 to 95 years (mean, 54 years). Follow-up ranged from 1 to 288 months (mean, 48 months). DLBCL was the most common histological diagnosis (53%). Treatment by mastectomy offered no survival benefit. Treatment that included radiation therapy in stage I patients (node negative) showed benefit in both survival and recurrence rates. Treatment that included chemotherapy in stage II patients (node positive) showed benefit in both survival and recurrence rates. Disease-free survival was 44.5% overall. In conclusion, mastectomy offers no benefit to the treatment of PBL. Nodal status predicts outcome and guides the optimal use of radiation and chemotherapy.

Treatment of primary breast lymphomas follows treatment recommendations for lymphomas of the same stage and histology in other locations. The choice of chemotherapeutic regimen should be based upon histological subtype, disease extent and the individual patient.

In 2008, Ryan et al.*.*[6] published the results of a retrospective international study of 204 eligible patients with the PBL DLBCL histological subtype attending International Extranodal Lymphoma Study Group-affiliated institutions from 1980 to 2003. There was no benefit from mastectomy, as opposed to biopsy or lumpectomy. Anthracycline-based chemotherapy was associated with higher overall survival (OS). Median OS was 8.0 years, and median progression-free survival (PFS) was 5.5 years. In conclusion, the combination of limited surgery, anthracycline-containing chemotherapy and involved-field radiotherapy produced the best outcome in the pre-rituximab era. At a median follow-up time of 5.5 years, 37% of patients had progressed; 16% in the same or contralateral breast, 5% in the central nervous system and 14% in other extranodal sites.

A similar opinion was expressed by Aviles et al.*.*[20] on the basis of their prospective study in which 96 patients were allocated to radiotherapy with a total dose of 45 Gy (*n* = 30), chemotherapy (6 × CHOP-21) (*n* = 32) and combined modality treatment (6 cycles of CHOP and radiotherapy with a total dose of 30 Gy) (*n* = 34). All included patients were in the early stage (I or II according to the Ann Arbor criteria) of PBL, most of them within an intermediate IPI score risk. At 10 years, the actuarial OS was 50, 50 and 76% in the treatment groups, respectively [20]. The authors postulated that combined therapy was the best method of treating patients with PBL. In our study, only two patients were treated with combined modality treatment with a complete response.

The impact of adding rituximab to chemotherapy has not been studied for primary breast lymphoma*.* In single arm prospective study [21], Aviles et al. evaluated impact of adding Rituximab to chemotherapy. Patients with early stage DLBCL-PBL were allocated to receive six cycles of CEOP-R chemotherapy (cyclophosphamide, epirubicin, vincristine, prednisone and rituximab) with granulocyte colony stimulating factor (G-CSF). There were no differences in either the CR (87%) or EFS (63%) rates compared to the patients described in their previous study [20].

Although the combination of anthracycline-based chemotherapy with rituximab should be considered standard, it appears that the addition of rituximab improves the outcome of all clinical and molecular subtypes of CD20-positive diffuse large B-cell lymphoma [22-24].

The role of central nervous system (CNS) prophylaxis in DLBCL of the breast is controversial. There have been no prospective trials of CNS prophylaxis in this population. Case series have reported a high incidence of CNS recurrence, estimated at 12 to 27%. Nevertheless, given this high incidence of CNS recurrence, central nervous system (CNS) prophylaxis should be considered [6,19,25].

Jeanneret-Sozziet al*.*[25] performed a multicenter international retrospective analysis on PBL within the Rare Cancer Network to assess the clinical profile, treatment outcome and prognostic factors in primary breast lymphoma (PBL). Between 1970 and 2000, 84 consecutive patients with PBL were treated at 20 institutions. Forty-six patients had Ann Arbor stage IE, 33 stage IIE, 1 stage IIIE, 2 stage IVE and 2 an unknown stage. Twenty-one underwent a mastectomy, 39 conservative surgery and 23 biopsy; 51 received radiotherapy (RT) with (*n* = 37) or without (*n* = 14) chemotherapy. Median RT dose was 40 Gy. Following this treatment, 10 (12%) patients progressed locally, and 43 (55%) had a systemic relapses. The central nervous system (CNS) was the site of relapse in 12 (14%) cases. The 5-year overall survival, lymphoma-specific survival, disease-free survival and local control rates were 53%, 59%, 41% and 87%, respectively. Local control is excellent with RT or the combined modality treatment, but systemic relapses, including in the CNS, occur frequently.

In Rayan et al.’s patients [6], one unexpected finding was that the risk of CNS relapse was relatively low, occurring in only 5% of patients. This is at variance with the reports of other smaller studies and is considerably lower than the risk seen in primary testicular DLBCL. It may be that primary breast DLBCL does not have the same tropism for CNS as testicular DLBCL, and this difference explains the generally superior survival for patients with primary breast DLBCL compared with that of patients with primary testicular DLBCL. However, it is possible that limiting the eligibility to patients with localized disease has led to underestimation of the rate of CNS involvement. This result should be considered with caution. In conclusion, the authors suggested using prophylaxis to avoid central nervous system involvement.

A review of the literature is presented in Table 3.

**Table 3:** Literature review

**Authors**	**Type of study**	**Number of patients**	**Histology**	**Treatment**	**Follow-up (years)**	**Survival**
Jennings et al. 2007	Database analysis	465	DLBCL, follicular lymphoma, MALT	Mastectomy +/− Chemotherapy +/− Radiotherapy	4	DFS 44.5% overall, no benefit from mastectomy
Ryan et al., 2008	Retrospective	204	DLBCL, MALT	Surgery, chemotherapy, radiotherapy, combined therapy	5.5	Median OS: 8 years, Median PFS: 5.5
Aviles et al., 2005	Prospective	96	DLBCL	RT (45 Gy)	10	CR: 66%, EFS: 50%, OS: 50%
				CHOP-21 × 6		CR: 59%, EFS: 57%, OS: 50%
				CHOP × 6/RT		CR: 88%, EFS: 83%, OS: 76%
Aviles et al., 2007	Prospective Single arm	32	DLBCL	CEOP-R 14 × 6	5.3	CR: 87%, EFS: 63%
Jeanneret- Sozzi et al., 2008	Meta- analysis	84	High-, intermediate-, low-grade lymphomas	Surgery +/− Radiotherapy +/− Chemotherapy	4	OS 5 years 53%, DFS 41% local control rate 87%

*DLCL* diffuse large cell lymphoma; *MALT* mucosa-associated lymphoid tissue lymphoma; *CR* complete remission; *CHOP* cyclophosphamide, doxorubicin, vincristine, prednisone, *CEOP* yclophosphamide, epirubicin, vincristine, prednisone; *R*: rituximab; RT: radiotherapy; *OS* overall survival; *EFS* event-free survival; *DFS* disease-free survival.

## Conclusions

Primary malignant lymphoma of the breast (PLB) appears to be a rare disease, and few clinicopathological features of the disease have been discussed in prior studies. Our series contained a small sample size, but it is interesting because it included only DLBCL cases, one case of male breast lymphoma and one of bilateral breast lymphoma.

No clear consensus concerning the therapy has emerged, although chemotherapy seems to be the more common choice, alone or in combination with other treatments. The choice of chemotherapy regimen and/or use of radiation therapy (RT) is based upon the histological subtype, disease extent and individual patient. The prognosis and effectiveness of individual treatments are generally extrapolated from therapy for other extranodal lymphomas.

## Abbreviations

PBL, Primary breast lymphoma; NHL, Non-Hodgkin’s lymphoma; DLBCL, Diffuse large B-cell lymphoma; RT, Radiotherapy; IELSG, International Extranodal Lymphoma Study Group; MALT, Mucosa-associated lymphoid tissue lymphoma; CNS, Central nervous system; CHOP, Cyclophosphamide, doxorubicin, vincristine and prednisone; CEOP-R, Cyclophosphamide, epirubicin, vincristine, prednisone and rituximab; PNET, Tumeurs Neuroectodermiques Périphériques Primitives; 5 FU, 5-fluoro-uracile; ICE, Ifosfamide, carboplatin and etoposide; CR, Complete remission; OS, Overall survival; PFS, Progression-free survival.

## Competing interests

The authors declare that they have no competing interests.

## Authors’ contributions

MB drafted the manuscript. All authors read and approved the final manuscript. All authors read and approved the final manuscript.
