# Correction: Primary malignant non-Hodgkin’s lymphoma of the breast: a study of seven cases and literature review

**DOI:** 10.1186/1477-7819-10-217

**Published:** 2012-10-11

**Authors:** Bourhafour Mouna, Boutayeb Saber, El Harroudi Tijani, M’rabti Hind, Taleb Amina, Errihani Hassan

**Affiliations:** 1 Department of Medical Oncology, National Institute of Oncology, Rabat, 10000, Morocco; 2 Faculty of Medicine, Oujda, Morocco, Department of Surgery, National Institute of Oncology, Rabat, 10000, Morocco

## Correction

After publication of this work [1], we noted that we had inadvertently included Bodmer Alexandre, Castiglione-Gertsch Monica and Pierre Yves Dietrich as authors of the study. The list of authors has now been corrected and the Authors' contributions and Competing interests section modified accordingly.

## Competing interests

The authors declare that they have no competing interests.

## Authors’ contributions

MB drafted the manuscript. EH T participated in the design of the study. MB reviewed the final manuscript and revised it critically for important intellectual content. All authors read and approved the final manuscript.
